# First Medical Examining Board

**Published:** 1898-01

**Authors:** William Warren Potter


### Special Article.

### FIRST MEDICAL EXAMINING BOARD.

REPRESENTING THE MEDICAL SOCIETY OF THE STATE OF NEW YORK.

ON the 5th day of June, 1890, the governor approved the act of the legislature creating three separate medical examining and licensing boards—one to represent the medical society of the state of New York, one to represent the homeopathic medical society of the state of New York, and one to represent the electic medical society of the state of New York. It was provided that the law should take effect September 1, 1891, and that prior thereto these several state medical societies should each nominate fourteen or more members to the regents of the University of the state of New York, from which number the regents were to appoint three state boards of examiners, each to consist of seven members.

On March 11, 1891, the regents appointed to represent the medical society of the state of New York the following-named examiners: For three years, Dr. William Warren Potter, of Buffalo, Dr. William S. Ely, of Rochester, and Dr. Maurice J. Lewi, of Albany; for two years, Dr. William C. Wey, of Elmira, and Dr. George Ryerson Fowler, of Brooklyn; for one year, Dr. Eugene Beach, of Gloversville, and Dr. Joseph P. Creveling, of Auburn. Since his appointment Dr. Lewi has removed to New York. These appointees met at Albany, September 1, 1891, and organised as a board of medical examiners by electing Dr. William C. Wey, president, and Dr. Maurice J. Lewi, secretary.

These examiners have been reappointed to fill their own vacancies whenever they occurred by expiration of terms, and the officers have been reëlected each year, so that the organisation has been one and the same from the beginning until the death of Dr. Wey, which occurred June 30, 1897. The regents have lately appointed Dr. A. Walter Suiter, of Herkimer, to be a member of the board vice William C. Wey, deceased. A group portrait of the board as first constituted is herewith presented and the following brief biographical sketches are appended, in the belief that the interest in the subject of improved medical education is sufficient to justify a record in accessible form that may be preserved and referred to, of some of those who have been especially instrumental in establishing the present system of separate state examination for license to practise medicine.

WILLIAM C. WEY was born at Catskill, January 12, 1829. After obtaining his preliminary education he became a pupil of Dr. Alden March and took his doctorate degree from the Albany medical college, January 23, 1849. He then located at Elmira and was its first health officer after it became a city, to which office he was again appointed in 1894. He was a member of the board of education for six years and a manager of the state reformatory over twenty-two years, having been appointed March 5, 1875. For a number of years he was one of the managers of the state inebriate asylum at Binghamton. He was a manager and senior consultant of the Arnot-Ogden memorial hospital ; a member of the Elmira academy of medicine, of the medical society of the county of Chemung and of the medical society of the state of New York, of all of which he had been president. He was chairman of the special committee on revision of the code of ethics of the medical society of the state of New York. He was appointed a member of the state medical examining and licensing board March 11, 1891, and was elected president at its first meeting, September 1, 1891. He was also examiner in physiology and hygiene, serving in this capacity as well as president from the organisation of the board until his death, which occurred at Elmira, where he had continued to reside for forty-eight years, on June 30, 1897.

WILLIAM S. ELY was born at Rochester in 1841, received his preliminary education in that city and took his degree in arts at the university of Rochester in 1861. He began the study of medicine immediately and during the civil war served as assistant surgeon 108th regiment N. Y. Vols. from August 13, 1862, to September 2, 1863, when he was commissioned assistant surgeon U. S. Vols. He was promoted to be surgeon U. S. Vols., January 20, 1865. He was mustered out at his own request October 6, 1865, and was breveted lieutenant-colonel U. S. Vols., for faithful and meritorious service. He received his doctorate degree from the college of physicians and surgeons at New York in 1867, since which time he has practised his profession in his native city. He was appointed attending physician to the Rochester city hospital, in 1868, and still continues in that service. He is a member of the Rochester medical society, of the medical society of the county of Monroe, of the medical association of central New York, of the medical society of the state of New York, of all of which he has been president. He was a member of the special committee on revision of the code of ethics of the medical society of the state of New York, his colleagues being Drs. Wey, Agnew,

Vander Poel and Piffard. He was appointed by the regents, March 11, 1891, to be a member of the state medical examining board and was reappointed in 1894 and 1897. He is, and has been from the first, examiner in anatomy.

Eugene Beach was born at Greenville, Greene county, N. Y., November 6, 1838. He obtained his preliminary education in the state normal school at Albany, from which he graduated in 1856. He received his doctorate degree from the Long Island college hospital, Brooklyn, in June, 1866, and soon afterward began the practice of his profession at Gloversville, which he has continued until the present time. He is a member of the medical society of the county of Fulton and of the medical society of the state of New York and is attending physician to the Nathan Littauer hospital at Gloversville. He was appointed by the regents, March 11, 1891, to be a member of the state medical examining board and was reappointed in 1892 and 1895. He became at the outset, and still continues in that position, examiner in the practice of medicine, materia medica and therapeutics.

George Ryerson Fowler was born in New York City, December 25, 1848, and graduated from Bellevue hospital medical college in February, 1871. He was one of the founders of the Brooklyn anatomical and surgical society in 1878, and was associate editor of the *Annals* of this society, now the *Annals of Surgery*. In 1883 he was appointed surgeon-in-chief to the department of fractures and dislocations at St. Mary's hospital, Brooklyn, and subsequently had entire charge of the general surgery of that hospital. He has been surgeon to the Methodist Episcopal hospital since its opening in 1887. He was president of the medical society of the county of Kings in 1886 ; in 1891 was elected a fellow of the American surgical association, and in 1892 a member of the New York surgical society. He is a member of the New York academy of medicine ; one of the founders of the Brooklyn surgical society and its president in 1891, and a permanent member of the medical society of the state of New York.

In 1895 he was appointed to service at the Brooklyn hospital, of which he is now surgeon-in-chief. He is the author of a treatise on Appendicitis and of many surgical monographs.

Dr. Fowler was appointed by the regents, on March 11, 1891, a member of the state medical examining board, was reappointed in 1893 and 1896, and has been from the first and is now examiner in surgery. He is also a member of the question committee.

Joseph P. Creveling was born at Bethlehem, N. J., May 2, 1848. After receiving his preliminary education he pursued the study of medicine and received his doctorate degree from the

University of Michigan in 1870. Later the honorary degree of master of arts was conferred upon him by Maryville college. He has practised the profession of medicine since 1872 at Auburn. He is a member of the medical society of the county of Cayuga, of the medical association of central New York, and of the medical society of the state of New York. He is attending surgeon at the Auburn city hospital, and has held several other places of honor and trust. March 11, 1891, he was appointed by the regents a member of the state medical examining board, and upon its organisation was assigned to the department of pathology, which he has continued to hold from the first. He was reappointed in 1892 and 1895.

Maurice J. Lewi, son of Dr. Joseph Lewi, was born at Albany, N. Y., December 1, 1857. After graduating from the Albany free academy (high school) and taking a private Cornell course with the late Professor Altmayer, he entered the Albany medical college, graduating in 1877. After serving as house physician at the Albany hospital he went to Europe for study and returning to Albany he commenced the general practice of medicine. He was appointed lecturer at the Albany medical college on diseases of women and children, in which position he continued for two years and in 1885 he became professor of medical jurisprudence at the Albany law school. He was president of the Albany county medical society, the Albany academy of medicine and the union medical association. He removed to New York City in 1892. He was appointed medical director of the American Union Life Insurance Company in 1895, which position he still occupies.

Dr. Lewi is a member of the society of medical jurisprudence, the medical society of the county of New York, the New York academy of medicine and the medical society of the state of New York. Dr. Lewi was appointed by the regents, March 11, 1891, a member of the state medical examining board and was reappointed in 1894 and 1897. He has been secretary of the board, examiner in chemistry and a member of the question committee from the first.

William Warren Potter was born at Strykersville, N. Y., December 31, 1838, obtained his preliminary education at Arcade seminary and Genesee seminary and college, Lima, N. Y., and graduated from the Buffalo university medical college in 1859. After two years of general practice he was commissioned assistant surgeon of the 49th regiment N. Y. Vols. (Col. D. D. Bidwell commanding), and in December, 1862, was promoted surgeon of the 57th regiment N. Y. Vols. In 1863, he was assigned as surgeon-in-chief of the first division hospital second army corps, and upon muster out of service at the close of the war was breveted lieutenant-colonel of U. S. Vols. (date March 3, 1865), for faithful and meritorious service. He is a permanent member of the medical society of the state of New York, president in 1891 ; member of the medical society of the county of Erie, president in 1893; was president of the Buffalo medical and surgical association in 1886 ; was president of the Buffalo obstetrical society, 1884-86 ; secretary of the American association of obstetricians and gynecologists since 1888 ; president of the section of gynecology and abdominal surgery of the first Pan-American medical congress, in 1893 ; president of the national confederation of state medical examining and licensing boards, 1895–'98 ; consulting gynecologist at the woman's hospital, Buffalo ; and a companion of the military order of the loyal legion of the United States.

Dr. Potter was appointed a member of the state medical examining board by the regents March 11, 1891, and was reappointed in 1894 and again in 1897. He has been examiner in obstetrics from the organisation of the board until the present time.

Examinations have been held by the board as constituted in the foregoing paragraphs since the autumn of 1891, five times a year simultaneously in four cities—namely, New York, Albany, Syracuse and Buffalo. The board makes an annual report of its work to the medical society of the state of New York, and detailed reports of each examination are made to the regents. These latter are very elaborate tables, showing the percentages in standing of each candidate in each branch and his aggregate standing as a whole. These statistics involve an extraordinary amount of labor in their preparation, since they must be made up with the utmost care. This work devolves upon the secretary, whose office of necessity is a busy one. Under the stimulating influence of the state examination medical colleges have advanced their standards and lengthened their periods of instruction until, beginning with 1898, candidates for the state license must present a diploma from a schoolr equiring four years of collegiate training and evidence of a preliminary education equal to that of a New York high school graduate.

The medical profession of the state of New York has occasion to feel justly proud of its standards of medical education as at present existing.

